# Roles, barriers and behavioral determinants related to community pharmacists' involvement in optimizing opioid therapy for chronic pain: a qualitative study

**DOI:** 10.1007/s11096-021-01331-1

**Published:** 2021-10-02

**Authors:** Aziza Alenezi, Asma Yahyouche, Vibhu Paudyal

**Affiliations:** School of Pharmacy, Institute of Clinical Sciences, College of Medical and Dental Sciences, Sir Robert Aitken Institute for Medical Research, University of Birmingham, Edgbaston, Birmingham, B15 2TT UK

**Keywords:** Chronic pain, Community pharmacists, Medicine optimization, Opioid, Qualitative, Theoretical domains framework

## Abstract

*Background* Opioid are currently widely used to manage chronic non-malignant pain (CNMP), but there is a growing concern about harm resulting from opioid misuse and the need for medicine optimization, in which pharmacists could potentially play a key role. *Objective* This study explored pharmacists' roles, barriers and determinants related to their involvement in optimizing prescribed opioids for patients with chronic pain. *Setting* Community pharmacies in the United Kingdom. *Method* Semi-structured interviews based on the Theoretical Domains Framework were conducted between January and May 2020 with 20 community pharmacists recruited through professional networks. Data were analysed thematically. *Main outcome measure:* Pharmacists’ perceived roles, barriers and behavioural determinants in relation to opioid therapy optimization. *Result* Pharmacists demonstrated desire to contribute to opioid therapy optimization. However, they described that they were often challenged by the lack of relevant knowledge, skills and training, inadequate time and resources, systemic constraints (such as lack of access to medical records and information about diagnosis), and other barriers including relationships with doctors and patients. *Conclusion* The contribution of community pharmacists to optimize opioid therapy in CNMP is unclear and impeded by lack of appropriate training and systemic constraints. There is a need to develop innovative practice models by addressing the barriers identified in this study to enhance the contribution of community pharmacists in optimization of opioid therapy for chronic pain.

**Supplementary Information:**

The online version contains supplementary material available at 10.1007/s11096-021-01331-1.

## Impacts on practice

- Pharmacists are keen to contribute to opioid optimization by educating patients, managing their expectations, monitoring and referring problems, as part of a patient-centred approach.
- Constraints impeding community pharmacists' potential role in prescribed opioid optimization such as lack of training, and system-related barriers should be addressed.

## Introduction

Chronic pain is defined as pain that persists for more than three months [1]. The World Health Organization (WHO) data suggest that the prevalence of chronic pain is 22% of the world population [2] and in the UK 46% of the population suffer from chronic non-malignant pain (CNMP) [3]. The concerns about prescribed opioid and chronic opioid therapy (COT) effectiveness, safety, and abuse in CNMP have grown, especially with the alarming death rate related to medical and illicit use of opioid in North America [4]. The extent of the increased opioid use, including misuse, remains less debated in the UK. Yet, UK based studies indicate an upward trend in the use of prescribed opioid in the National Health Services (NHS) parallel to that in the US [5].

Medicine optimization, which means ensuring safe, effective, and efficient medicine use, is becoming a key priority in the UK's NHS to manage long-term conditions [6]. Community pharmacists have a role in optimizing prescribed opioid use in CNMP patients and ensuring safe, effective opioid use as well as making sure that these patients are benefiting from using these medications.

Findings from literature suggest that a well-structured CNMP management program led by a multidisciplinary team is critical to optimize COT use and improve CNMP patients' quality of life [7]. One of the most important services that patients with chronic diseases receive at community pharmacies in the UK is Medicine Use Review (MUR) which has been performed since 2005. MUR is a 'concordance/compliance review' and annual face-to-face patient–pharmacist consultation for patients using two or more prescribed medicines and are regular customers of the pharmacy [8]. Through such reviews and other patient contact, community pharmacists are well-positioned to deliver many services that can lead to optimization of opioid use in CNMP [9].

A systematic review (2020) found that community pharmacists faced various challenges when trying to play the role of opioid therapy optimizer [10]. The first set of challenges is related to patients, including demand for early refill, unrealistic expectations of pain relief and inadequate access to non-pharmacological treatment. There are also difficulties dealing with patients on high doses of opioid. The second set of challenges is pharmacist-related challenges such as lack of training, lack of confidence, lack of access to medical records and lack of guidelines and policy that integrate pharmacists with other healthcare providers in managing CNMP [10]. Other studies from the USA and the UK suggest that community pharmacists have constructive attitudes toward expanding their role in optimizing opioid use and pharmacy-based screening and intervention to reduce substance use disorder in patients with CNMP [9, 11].

There are, however, gaps in the literature in community pharmacists' contribution to opioid optimization in the UK. Most existing UK studies focus on pharmacists' role regarding over-the-counter medication. This may be because the extent of the opioid crisis in the UK is different to those in USA, Canada and Europe, where other studies have been done [9, 12]. To the authors ‘knowledge, this is the first qualitative study of UK pharmacists' regarding their roles, associated barriers and and behavioural determinants regarding their roles in opioid therapy optimization.

### Aim of the study

The aim of this study was to explore community pharmacists' perceptions regarding their roles, barriers and determinants related to their involvement in optimizing prescribed opioids CNMP.

### Ethics approval

Ethical approval was granted by the Science, Technology, Engineering and Mathematics Ethical Review Committee of the University of Birmingham (ERN_19-1922). Written informed consent was obtained from all participants.

## Method

### Study design

This study was reported based on the COREQ (Consolidated Criteria for Reporting Qualitative Research) checklist for comprehensive reporting of qualitative studies [13] (see Additional file 1). Data were collected between January and May 2020 via semi-structured interviews informed by the Theoretical Domains Framework (TDF). The TDF was developed from a synthesis of psychological theories to help apply theoretical approaches to behaviour change interventions. TDF domains represent environmental, cognitive, and social factors that may affect behaviour [14, 15]. The TDF has been widely used to understand behaviours and implementation challenges in various settings and content areas in health care, including community pharmacies [16, 17] and hence is suitable in the context of this research.

### Sample

**Fig. 1:**
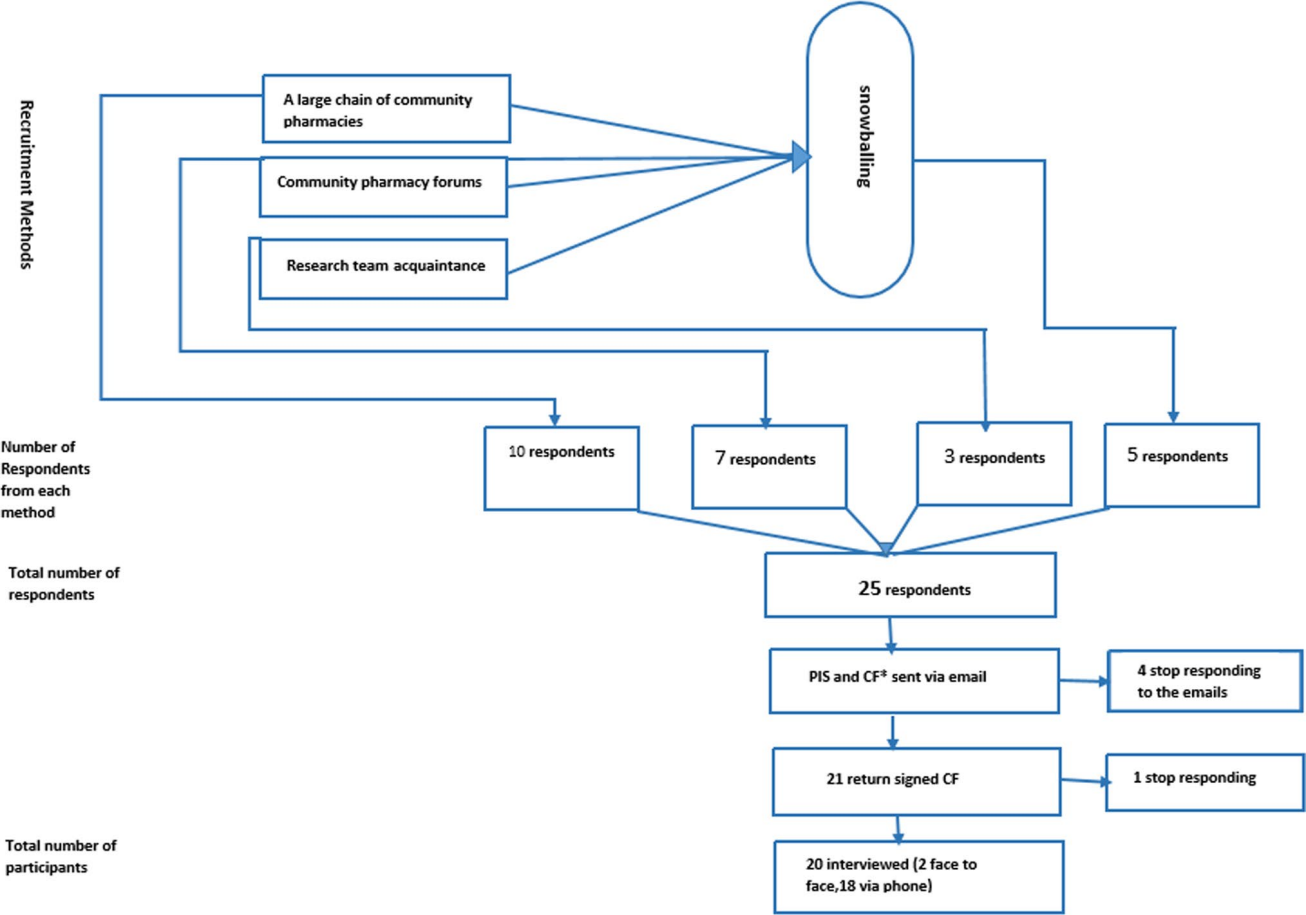
Study sample recruitment flowchart. *Note* PIS: Participant Information Sheet, CF: Consent Form

Community pharmacists working in various regions (cities and rural areas) in England were recruited through the dissemination of invitation using the professional networks of the research team, including community pharmacy forums, a large chain of community pharmacies in England and acquaintance of the research team. The inclusion criterion was currently working in community practice, and there were no exclusion criteria. The primary researcher (AA) introduced the study to the pharmacist and asked if they would consider taking part in the study. A 'snowballing' sampling method was used to recruit more participants, whereby interviewed pharmacists identified others who might be interested in taking part. See Fig. 1.

### Interview topic guide and procedure

The TDF informed the development of an interview guide containing open-ended questions and standardized prompts available for use if needed. It was reviewed within the research team and pilot tested with two community pharmacists. However, some questions under specific domains such as intention were considered repetitive or irrelevant by the participants and were deleted in the following interviews. However, each domain was represented by at least one question. (See Table 1). Written information about the study was offered (by email) before the interview. An agreed interview date, time, and location (for face-to-face interviews) were then arranged. All interviews were conducted by AA. Face-to-face interviews were at the pharmacist's place of work. Telephone interviews (when lockdown in response to COVID-19 prevented face-to-face interview) were conducted in the researcher's home in a quiet room, at a time convenient to the participant. No follow up interviews were conducted. Interviews ranged in duration from 25 to 65 min, mostly taking around 35 min. All interviews were audio-recorded and anonymously transcribed verbatim. Recruitment and interviewing ceased after reaching thematic saturation based on initial analysis and coding (no new themes or codes could be identified from the last two interviews) [18].

**Table 1:** Mapping of interview schedule to TDF domains

Domain	Content	Sample questions as applied to this study
Knowledge	An awareness of the existence of something	What does chronic opioid therapy optimization mean to you? What are the knowledge barriers to optimizing opioid therapy for chronic pain?
Skills	An ability or proficiency acquired through practice	When considering filling a prescription for an opioid, how do you evaluate patient risk for misuse? What training have you had in how to appropriately review opioid prescription and identify opioid-related abuse and misuse?
Social/professional role and identity	A coherent set of behaviours and displayed personal qualities of an individual in a social or work setting	What are your general thoughts about dispensing opioid prescriptions?
Beliefs about capabilities	Acceptance of the truth, reality, or validity about an ability, talent, or facility that a person can put to constructive use	How can pharmacists contribute in recognizing the various forms of opioid abuse and misuse and in preventing inappropriate prescribing and diversion of opioids?
Optimism	The confidence that things will happen for the best or that desired goals will be attained	How optimistic are you that the use of e-prescription or prescription monitoring programmes will reduce inappropriate use of opioids?
Beliefs about consequences	Acceptance of the truth, reality, or validity about outcomes of a behaviour in a given situation	What are the advantages and disadvantages of opioid prescription review for each patient/ education about opioid to identify problematic medication use?
Reinforcement	Increasing the probability of a response by arranging a dependent relationship, or contingency, between the response and a given stimulus	How does your experience (good or bad) of reviewing opioid prescriptions/ education about the opioids in the past influence whether or not you would do it again?
Intentions	A conscious decision to perform a behaviour or a resolve to act in a certain way	Do you intend to review each opioid prescription and assess misuse and abuse? Why do you feel this way?
Goals	Mental representations of outcomes or end state that an individual wants to achieve	Do you think optimizing chronic opioid therapy is a personal goal or an institutional goal? What factors may interfere with the goal?
Memory, attention, and decision processes	The ability to retain information, focus selectively on aspects of the environment and choose between 2 or more alternatives	What reasons might prompt you to decide to carefully assess opioid-related abuse and misuse and review an opioid prescription?
Environmental context and resources	Any circumstance of a person's situation or environment that discourages or encourages the development of skills and abilities, independence, social competence, and adaptive behaviours	To what degree do resources and physical context influence your careful review of opioid prescription /education about the opioids?
Emotion	A complex reaction pattern involving experiential, behavioural, and physiological elements, by which the individual attempts to deal with a personally significant matter or event	How confident are you when dispensing high dose opioid prescription? How does this feeling influence what you do?
Social influences	Any circumstance of a person’s situation or environment that discourages or encourages the development of skills and abilities, independence, social competence, and adaptive behaviour	Tell me about your communication with prescribers regarding opioid prescriptions. When you need to contact the prescriber, are they accessible? What do you do if you cannot reach the prescriber?
Behavioural regulation	Anything aimed at managing or changing objectively observed or measured actions	What additional strategies do you think might mitigate inappropriate opioid use among CNMP patients?

### Qualitative data analysis

Data were analysed using the Framework method. Through listening to recordings and reading transcripts, a familiarisation process took place, from which AA attained an overview of specific beliefs within the data [19]. Following this step, excerpts from the interview transcripts were coded into one or more of the 14 TDF domains. Data were coded systematically using a deductive approach, whereby each TDF-14 domain served as a coding category [20]. Initially, a second researcher (VP) coded two randomly selected transcripts to ensure the credibility and dependability of the data analysis. Disagreement in coding between the two researchers was resolved through discussion with another research team member (AY) until consensus was reached. The team agreed on the coding framework and subsequent interview coding were regularly discussed in the team to ensure that they were consistent. Coded interview excerpts were compared and organized into a framework table generated in Microsoft Word®. When several codes could form a meaningful unit, this was deemed a theme [21]. A domain was deemed relevant if excerpts were coded frequently or if the participants emphasized the significant impact of behavioural determinants within a domain on their involvement in COT optimisation. A content analysis of the framework table was then undertaken to identify subthemes. Themes that shared the same central concept as the theme, but particularly important for the research question where consider a sub-theme. For example,’e-prescription’ was considered as subtheme under the umbrella of ‘Environmental Context’. Subthemes within each domain were summarised to give an overall impression of how each domain may influence the optimisation of prescribed opioid use for CNMP in the community pharmacy setting and illustrated using supporting quotations.

### Research trustworthiness

This study is part of AA's PhD studies after being trained in conducting research at the University of Birmingham. The research trustworthiness was sought by applying Lincoln and Guba's principles [22]. Credibility was enhanced through the adoption of appropriate, well-recognized research methods informed by previous comparable studies. Data analysis and coding were conducted by two researchers (AA, VP) [23]. To facilitate transferability, the author described the findings and supported the descriptions with quotes from the interviews. To enhance dependability and confirmability, the Theoretical Domains Framework was followed in conducting this study [24, 25].

## Results

Participants’ distribution and characteristics are presented in Tables 2 and 3.

**Table 2:** Distribution of participants (N = 20)

Location	N
London	2
Birmingham	2
Bradford	2
Leicester	2
Hull	2
Stourbridge	1
Solihull	1
Normanton	1
Coventry	1
Wakefield	1
Hemel hempstead	1
Sunbury-on-Thames	1
Missing information	3

**Table 3:** Sample characteristics (N = 20)

Characteristics	N
*Speciality and role*	
Community pharmacist	16
Community pharmacist and manager	1
Community and hospital pharmacist	3
*Sex*	
Male	17
Female	3
*Age in years*	
25–35	16
36–45	3
46–60	1
*Years of experience*	
1–5	11
6–10	3
11–15	4
20 +	2

Key themes are presented below with illustrative quotes.

For the result summery, see Table 4.

**Table 4:** Theoretical domains relevant to community pharmacists’ role in prescribed opioid optimisation with illustrative quotations

Theme	TDF domains & non-TDF subtheme	Codes/constructs	Illustrative quotations
Pharmacists’ self- perception	Social and prof role and identity	Primary guardian of patient welfare	Obviously, I am a pharmacist, and so my interest is always in patient health and patient's outcome (Participant 2)
		Secondary role	We’re not involved in it directly… It would be totally the medicine management team at the surgery … “As a pharmacist, you would intervene by highlighting it to the doctor because the doctor is going to take the decision at the end of the day (Participant 17)
		Safeguarding action	I would say it is part of my job to check whether they are… the amount that he was taking, Oramorph, isn't going to cause him on overdose or a serious reaction with alcohol misuse (Participant 14)
Capabilities	Knowledge	Knowledge of condition/scientific rationale	Opioids are not for chronic use ……they’re only when needed….in a flare-up or acute pain (Participant 8)
	Skills	Skills development through training	We did cover the guidelines, but it was quite some time ago (Participant 17)
		Experience	Tell if someone's misusing but, that's just from experience… or like, you know, intuition kind of thing. You would usually know anyway (Participant 14)
	Beliefs about capabilities	Empowerment (lack of)	It's currently, it's impossible to monitor and optimize opioid, opioid treatments. (Participant 18)
Infrastructure and systemic constructs	Environmental context and resources	Barriers: Information	Without access to medical records …. that’d probably be a barrier. (Participant 19)
		Funding and resources	The, erm, funding, I think that it’s one of the things that they probably might need to kind of fund to sort of, as a service…. (Participant 3)
		Systems	There's nothing stopping that patient taking their prescription to another pharmacy (Participant 10)
	E-prescriptions		They might not have the time to …do proper checks to make sure… and they might just print it (Participant 5)
Personal factors	Social Influences	Inter-group conflict with GP	That, I think, [causes]frustrations with all community pharmacists because we don’t have, erm, I think professional to professional communication between prescribers and pharmacist, so …..patients have to come back the next day or a few days after ……if there is a single piece of information that we need to clarify or double-check. Sometimes……you have to call back two, three times, and you still can’t get an answer. The patients might have to take their prescription somewhere else…. I think there’s a huge barrier between pharmacists and prescribers (Participant 13)
		With patients	Obviously, if you have a bad experience with a patient, then sometimes you think twice about something, you know, doing something (Participant 11)
	Emotion	Empathy	If [I] find out that the patient is misusing other substances with the opioid ….I'd be worried about them ( Participant 4)
		Stress	Monitoring and talking to every patient and keeping tabs is going to be a very strenuous task, that would be very stressful ( Participant 6)

### Theme 1: the pharmacists' self-perception of their identity and social and professional role

A dominant theme throughout the interviews, arising from the TDF domain, ‘Social and Professional Role and Identity, was pharmacists' perception of themselves as guardians of patient welfare. Some expressed this in general terms, as an intrinsic part of their professional role. This is illustrated in the quote below.

Obviously, I am a pharmacist and so my interest is always in patient health and patients' outcome (Participant 2).

Others elaborated on particular safety concerns, including appropriate dosage, ensuring that patients achieve optimum benefit from their medication and do not suffer undesirable physical or mental consequences, and looking out for signs of risk. As one respondent commented,

I would say it's part of my job to check whether [patients] are, erm, abusing alcohol, or their Oromoph. The amount he is using isn't going to cause him overdose or a serious reaction (Participant 14)

When such concerns arose, they felt a responsibility to take action. Some viewed prescribed opioid optimization as a specific responsibility, but others viewed their role as secondary, with the clinician having the main role, compared to over-the-counter opioid-containing products, where pharmacists have a bigger role to play. One interviewee explained,

About over the counter selling of co-codamol but, for prescriptions, then we're not involved in it directly… It would be totally the medicine management team at the surgery … As a pharmacist, you would intervene by highlighting it to the doctor, because the doctor is going to take the decision at the end of the day (Participant 9).

Pharmacists highlighted specific activities they performed pursuant to their safeguarding role, particularly medicine use review (MUR) in which they asked about patients' use of the medication and any problems they encountered. At these interviews, they might also ask patients about their alcohol consumption and over-the-counter medications, but admitted that they did not routinely raise these issues, as expressed in the example below.

We used to have the medicine user review so, that was an excellent opportunity to sit down with the patient and ask if their pain is controlled, they are taking any medication (Participant 11).

Another safeguarding activity was the provision of education and advice, including warning of possible side effects. In the event of serious concerns, pharmacists reported deferring supply of medicine and referring to the prescriber, for example, to query the dose, highlight interactions with other medications, or report suspicions of a patient's misuse or abuse of prescribed medication or other substances. Some participants gave examples of situations where they might check to prevent any negative consequences.

I would say it is part of my job to check whether they are… the amount that he was taking, Oramorph, isn't going to cause him on overdose or a serious reaction with alcohol misuse. (Participant 14).

### Theme 2: capability

The theme ‘Capability’ is related to the competencies pharmacists possess that facilitate their role in prescribed opioid optimisation, arising from the TDF domains, ‘Knowledge’, ‘Skills’ and ‘Beliefs about Capabilities’.

In performing their role, pharmacists relied on their professional judgement around supply of prescribed opioid, drawing on knowledge and skills derived from training and experience. They displayed an understanding of the meaning of COT optimization as ensuring the balance between effective control of patients' condition and avoiding harmful side effects. They revealed knowledge of opioid-related problems, most of them mentioning dependence, tolerance and addiction, as well as physical and psychological side-effects such as depression. They also showed awareness of other treatment options, including non-opioid medication and complementary, non-pharmacological therapies that might reduce the need for opioid:

Opioids are not for chronic use they're only when needed...in flare up or acute pain (Participant 8).

However, the majority reported having little or no formal training on how to review opioid prescriptions and identify misuse; only "one lecture or something" (Participant 17) but "no sort of course" (Participant 15). Some, moreover, admitted that their knowledge might not be up-to-date, since they qualified "a very long time ago" (Participant 20). Nevertheless, they generally felt confident in dispensing opioid and also suggested that, over time, they acquired an "intuition kind of thing" (Participant 14) that helped them to identify warning signs of misuse, such as over-ordering, early re-ordering and taking multiple medications. Despite their knowledge, skills and experience, however, several interviewees were pessimistic about their capability to contribute effectively to COT optimisation, due to their lack of authority.

Since, we’re sort of almost the end point before the patient gets their medication, so apart from the medication check, there's very little I can do (Participant 15).

### Theme 3: infrastructure and systemic constraints

Pharmacists also highlighted specific constraints preventing them from playing a more direct and effective role in COT optimization. The TDF domain ‘Environmental Context and Resources’ constituted one of the largest bodies of responses in the data set and revealed the importance of lack of resources and systemic constraints in explaining the limited involvement of community pharmacists in COT optimization. For example, pharmacists spoke of lack of information, whether guidelines, detailed patient records, or statements of policy issued by the employing pharmacy or chain, to guide them in assessing prescription appropriateness or the possible need for an intervention. With regard to patient information, it was pointed out that, although pharmacists could access summary care records, in practice, they would not do it without a specific reason, and in any case, the information contained might be too brief to be really helpful. For example, one participant said,

It doesn't show the full history; it's only a brief summary (Participant 5).

Where guidelines existed, their effectiveness, it was suggested, was undermined by ambiguity or inconsistency,

Each area, each prescriber, you know, they will have different guidelines, different rules (Participant 1).

Pharmacists also pointed to a lack of funding and resources, especially lack of time, as a barrier to more effective prescribed opioid optimisation. They discussed the difficulty of finding time to review, monitor and educate the huge number of patients they encountered and perceived involvement in opioid optimization as an additional burden on an already heavy workload. As interviewees pointed out, devoting the necessary time to review each opioid prescription and having a detailed conversation with every patient would not be feasible without additional staff.

Pressure is building up and pharmacist "wouldn't be interested something that increase my workload **(**Participant 15)

Additionally, lack of funding was discussed as a particular issue for many. Several participants suggested that opioid prescription review needed to be a funded service, to persuade pharmacies to undertake it, and for pharmacists to devote their time to it. Currently, they reported, the number of MURs performed annually was being reduced for financial reasons, and each pharmacy received payment for 250 reviews instead of 400 as previously, with further reductions forthcoming. Moreover, the reviews were limited to specific categories of medication, which did not include pain management:

We used to focus on blood pressure medication, diabetics and the like, so pain wasn't among those (Participant 16).

Ideally, it was suggested, MURs should be conducted for a wider range of conditions and medications than at present and more frequently. One experienced pharmacist suggested the need for a facilitative tool such as a short questionnaire to assess alcohol intake, break-through pain, and the like, to guide pharmacists in collecting the information needed to inform optimization interventions. A large volume of comments focused on factors related to the system of prescription ordering and processing, particularly the difficulty created by repeat prescribing and prescription delivery services, which reduced contact between patient and pharmacist and, hence, opportunities for education and monitoring. An added difficulty, especially with over-the-counter medications, was patients' freedom with regard to the choice of pharmacy and number of pharmacies visited, potentially enabling them to engage in what one pharmacist likened to a "pub crawl" for medications, making it difficult to monitor their behaviour. Moreover, the development of e-prescriptions, a widely used component of prescribing systems, was seen as of limited value in monitoring and controlling opioid prescriptions and opioid use. Whilst it was acknowledged that e-prescriptions ensure legality, reduce the possibility of error and create a better audit trail, overall, it was suggested, they would make little difference, and they might make it too easy for patients to obtain repeat prescriptions without review, especially since it was feared that other healthcare providers might not do the proper check, as stated by one participant:

They might not have time to do proper checks of the prescriptions and just print it (Participant 17).

Some participants indicated that an electronic prescription drug monitoring program such as the Prescription Drug Monitoring Programs (PDMPs) would be useful in the detection of opioid abuse/misuse.They were aware of the limitation of other electronic programs and suggested that although these may be necessary, they are not adequate to solve the problem of patients going from one pharmacy to another.

I get there would be an electronic program, it would be good if it's regular patients, but what if someone goes from another pharmacy or place? (Participant 10).

This might highlight community pharmacists' perception of their relative powerlessness to monitor and control opioid prescribing or even, sometimes, knowledge of how that role is carried out.

### Theme 4: personal factors

Other perceived constraints, captured by the TDF domain, Social Influences, were said to stem from relationships with other healthcare providers and patients, whose roles, expectations and perceptions affected pharmacists' performance of their role. A key issue in this regard was the difficulty for some pharmacists of communication with prescribers in the event of any query. While some respondents indicated that doctors were usually accessible and that queries were usually answered within a reasonable time, others expressed frustration at the delays they experienced when they could not get through to the prescriber and were forced to hold a prescription until the required check could be made.

Sometimes you have to call back two or three times and you can't get on answer. There's a huge barrier between pharmacists and prescribers (Participant 11).

Pharmacists also reported difficulties in COT optimization arising from patients' attitude and understanding. They pointed out that patients are understandably concerned primarily with their symptoms and simply want relief from pain, so tend to be resistant to the idea of reduction and put pressure on pharmacists who, in turn, are wary of upsetting a patient. They also felt unable to intervene in a patient's right to make their own lifestyle choices. Nevertheless, they suggested that patients' complex individual needs require a gradual, personalized approach and frequent follow-ups, in which pharmacists might contribute. By helping to manage potential expectations and providing education on the advantage of deprescribing and limitation of medication, its correct use, potential risk and the role of medication as one component in an overall plan for pain management, they aspired to an ultimate aim of enabling patients to take more responsibility for their own pain management.

Among personal factors, Emotions, such as stress or empathy for patients, emerged as of limited relevance, being mentioned by only two interviewees. Instead, pharmacists were more concerned about the need to address the aforementioned infrastructural and systemic challenges in order to play an enhanced role as part of an integrated approach to the safety of patients using opioid.

## Discussion

Four themes were identified in relation to the community pharmacists' perceptions of their current and future roles, barriers and behavioural determinants around optimization of opioid therapy for CNMP. The majority of sub-themes fell under the TDF domains. The few exceptions reflected issues raised by pharmacists beyond the discussion of their own role, or those spanning more than one domain. Pharmacists expressed a belief that they had a limited role, if any, in relation to prescribed opioid optimization. They appeared to act as medication dispensers or patient educators rather than medicine optimizers. However, participants perceived themselves as gatekeepers capable of minimizing the misuse of opioid-containing products, consistent with the existing literature [26–28].

Nevertheless, their perception of doctors as the ultimate authority over patient care deterred pharmacists from contributing more to opioid optimization, as noted in previous studies.

[29–32]. Moreover, pharmacists of varying experience reported that opioid prescriptions, misuse and addiction were barely addressed in their training or continuing education. The findings align with previous studies arguing that there is inadequate education on pain management provided to medical students [33–35]. This suggests that training curricula need review in order to fit the NHS aim of giving the pharmacist an enhanced role in patient-centred care.

This study's findings suggested some pharmacists may be reluctant to be involved more in prescribed opioid optimization and screening for opioid misuse or abuse. This finding supports previous anecdotal reports of pharmacists' stress and discomfort when faced with a new responsibility, particularly in clinically complex, ambiguous and ethically sensitive situations. [33, 34]. Indeed, many participants viewed prescribed opioid optimization as not community pharmacists' responsibility and perceived they lacked the required authority, reimbursement, infrastructure and time for greater involvement in this area. Thus, for pharmacists to realize their potential in this area, improved resources and structures are needed to enhance clinical practice in the community setting [36].

Another perceived determinant of pharmacists' limited involvement with prescribed opioid optimization was the lack of regular communication with patients. This may be attributed to the growing use of online and mail-order pharmacies, as well as disability or limited mobility preventing CNMP patients from visiting the pharmacy [37]. Also, some participants mentioned that they did not discuss opioid risk, as they expected the prescriber to do so. This resulted in a situation where patients received no information about opioid's possible risks. Pharmacists could provide such information but may be deterred by the sensitivity of risk and problematic use issues [28]. More training and professional development regarding communication about sensitive issues could overcome this reluctance [38].

MUR services are designed to help pharmacists to discover medication-related problems. However, participants stated that even during MURs, prescribed opioid were not their priority. This may be attributable, in part, to a reported deficiency of patient information [39]. It would be reasonable for them to have access to diagnosis and information on co-morbidities to increase the clinical relevance of pharmacist recommendations and improve communications with other healthcare providers.

This study is not without limitations. Given the convenience sample involved in this study, findings may not be transferable. Also, topic sensitivity may have resulted in some response bias. The gender composition of the sample was skewed in favour of males and so may not reflect the actual gender balance in the profession. In addition, more than half of the sample had between 1–5 years' experience. Further research is needed to explore possible impacts of gender and experience on pharmacists' knowledge and capabilities toward opioid therapy optimization.

### Implications for practice

There is a need for a clearer definition and guidelines on the use of opioid for chronic pain and the optimization role that pharmacists can perform. Further education and training on pain and opioid use disorder are needed, consistent with the NHS vision of the expanded role of pharmacists in clinical aspects of patient care. There is also a need for direction and support from pharmacy organizations. For example, reimbursement would facilitate the expansion of pharmacists' role in patient care and encourage their involvement as opioid risk educators and gatekeepers.

### Further research

Further studies should explore the experiences and perceptions of pharmacists working in general practices and hospitals. In addition, the perspectives of wider stakeholders are imperative. This study is one of three interlinked complementary studies, the others exploring the perceptions of other CNMP healthcare providers and patients separately. The findings of the studies may be used to guide the development of future interventions and/or to inform policymakers of how to integrate the community pharmacists into opioid therapy optimization.

## Conclusion

The role of community pharmacists in optimizing opioid therapy for chronic pain was perceived by the study participants to be currently unclear. There is a potential to use community pharmacists' skills and knowledge to facilitate their involvement in treatment optimization, monitoring and educating patients. However, pharmacists perceived the need for appropriate training and tackling of systemic and resource constraints. There is a need to involve wider stakeholders in the developing innovative clinical pharmacy services allowing enhanced roles of pharmacists by addressing the barriers and behavioural determinants of further involvement identified in this study.

## Supplementary Information

Below is the link to the electronic supplementary material.

Supplementary file1 (PDF 548 kb)

## Data Availability

All data generated or analysed during this study are included in this published article.
